# Supplementary material for: Accurate high throughput alignment via line sweep-based seed processing
Source: Nat Commun. 2019 Apr 26;10:1939. doi: 10.1038/s41467-019-09977-2 (PMC6486643; doi:10.1038/s41467-019-09977-2)
Supplement: Supplementary file 1 — Supplementary Information [file 41467_2019_9977_MOESM1_ESM.pdf]

Accurate high throughput alignment via line sweep-based seed processing

Schmidt et al.

Supplementary Information

Supplementary Table 1 - Notations

Symbol	Description
$\sum_V$	Alphabet of sequence V
I_R	Reference index
$I_R^*(n)$	Suffix array initialization
$I_R^{\rightarrow}(c, O_{R,s})$	Forwards extension
$I_R^{\leftarrow}(c, O_{R,s})$	Backwards extension
$O_{R,s}$	Set of start indices of all occurrences of s in R
$\|V\|$	Length of the sequence V or size of the set V
$S \cup S'$	Union of the two sets S and S'
Q	Query sequence
R	Reference sequence
$V[0, p)$	Subsequence from 0 to p on the vector or sequence V
$V \oplus V'$	Concatenation of the elements or sequences V and V'
$s = (q, r, l)$	Seed s with query location q, reference location r and length l

The table shows the meaning of symbols used throughout the manuscript.

Supplementary Table 2 - Mean error rates

Name	Mean substitution rate	Mean insertion rate	Mean deletion rate	Location of sampled reads
PacBio	0.0382	0.0615	0.0277	ftp://ftp-trace.ncbi.nlm.nih.gov/giab/ftp/data/AshkenazimTrio/HG002_NA24385_son/PacBio_MtSinai_NIST/
UON	0.0774	0.0263	0.0301	ftp://ftp-trace.ncbi.nih.gov/giab/ftp/data/AshkenazimTrio/HG002_NA24385_son/Ultralong_OxfordNanopore/

The table comprises the sampled error rates for Pac Bio and UON reads. Additional details are given in Supp. Note 1.

Supplementary Fig. 1 - Length distributions

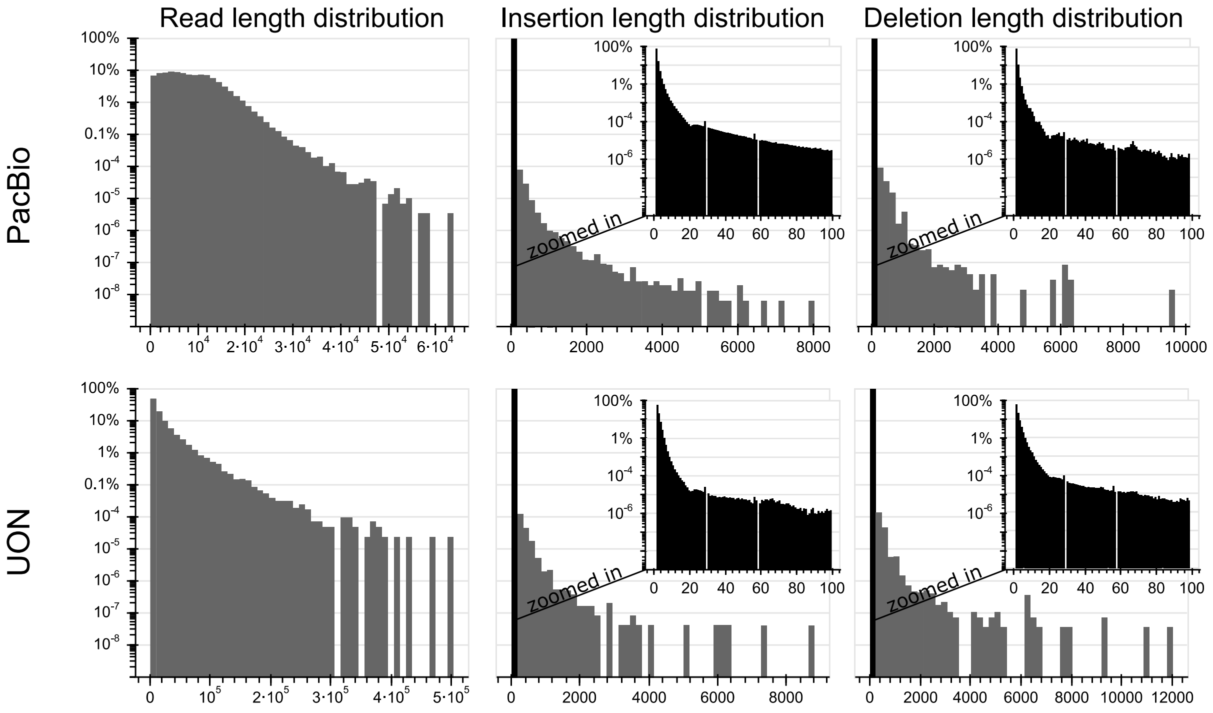

The figure shows the sampled distributions of insertions and deletions for PacBio and UON reads using Minimap2. More details are given in Supp. Note 1.

Supplementary Fig. 2 - Principal component analysis of simulated PacBio reads

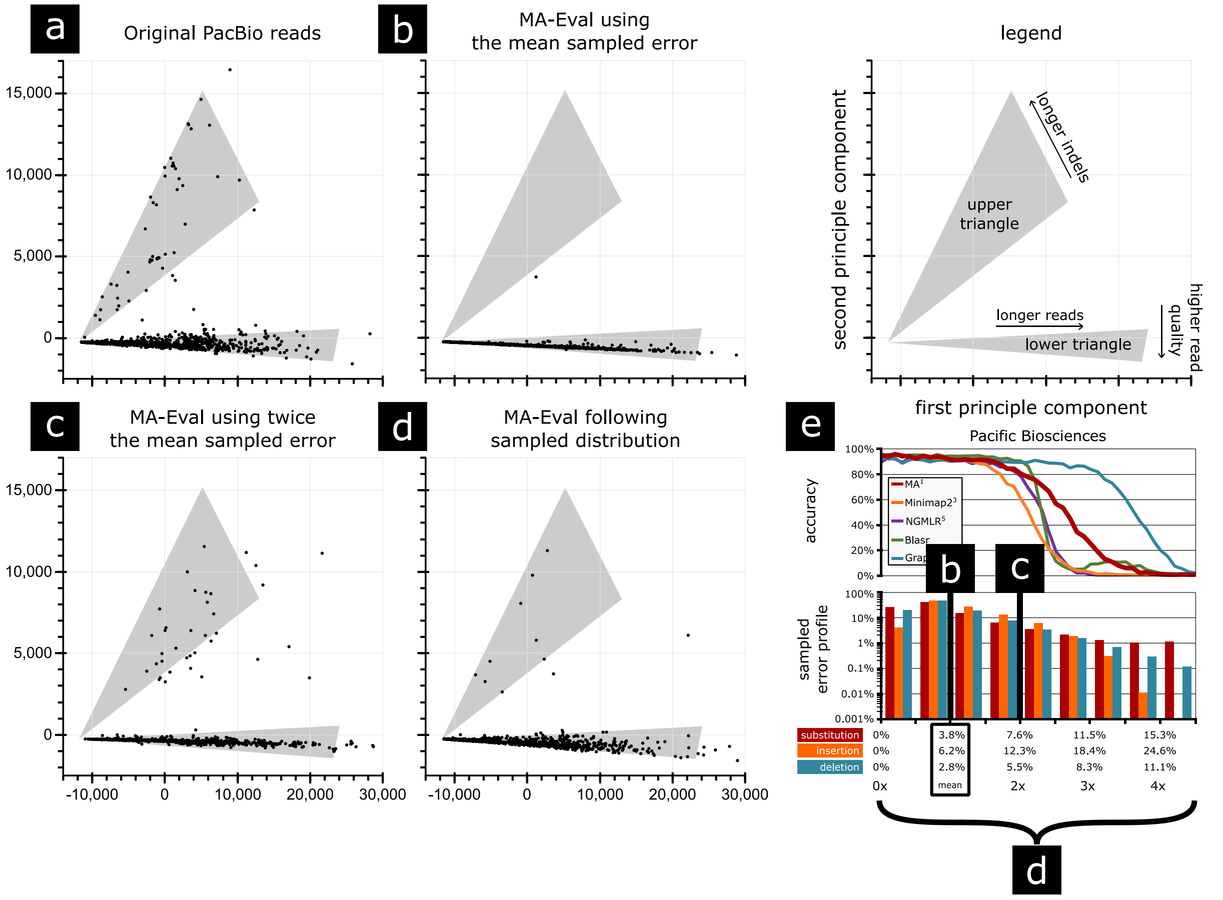

The figure shows the outcome of the principal component analysis (PCA) for our simulated PacBio reads. The dataset used for the PCA is reported in Supp. Table 2 (Location of sampled reads). In subfigure (a), which visualizes the original reads, the points are concentrated in two triangular areas. Subfigure (b) visualizes simulated reads using the mean error rates (for substitutions, insertions and deletions). The position labeled b in subfigure (e) shows the corresponding aligners' behaviors for these reads. In subfigure (c), the mean error rates are doubled.

The observed effects are:

1) The upper triangle gets populated.

2) The band in the lower triangle moves upwards and widens.

Accordingly, it gets more challenging for aligners to find the correct position of such reads, which is reflected by the declining accuracy for all aligners in subfigure (e).

Subfigure (d) visualizes the accumulative set of all reads for different error rates, where the sampled error profile follows the one given in subfigure (e) (individually for substitutions, insertions and deletions). A comparison of subfigure (a) and subfigure (d) shows that the lower triangle is well mapped by our accumulative approach, while the upper triangle is slightly underrepresented.

Supplementary Fig. 3 - Principal component analysis of simulated UON reads

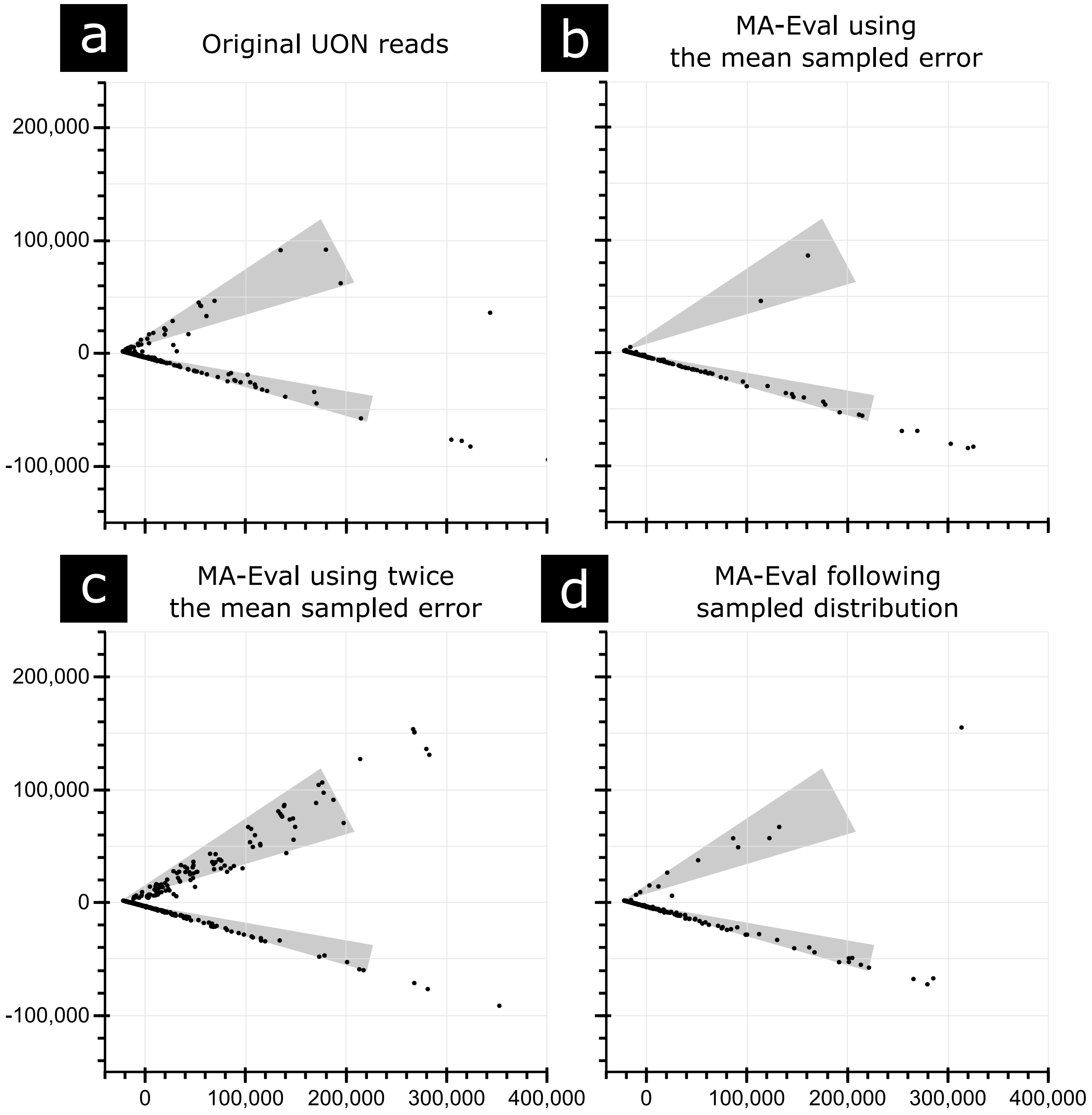

The figure shows the outcome of the PCA analysis for our simulated UON reads (the original reads are taken from the ftp source referred in Supp. Table 2). The analysis follows the scheme reported in Supp. Note 1. As for PacBio reads, two triangles get visible, where the lower triangle is more populated than the upper one. Compared to the PCA diagrams for PacBio reads, the lower triangle is thinner.

Supplementary Table 3 - PCA dimensions

Dimension	Name	Description
1	Amount mutations	Accumulated number of mismatches in cigar
2	Amount insertions	Accumulated number of insertions in cigar, where consecutive insertions are counted as one.
3	Amount deletions	Accumulated number of deletions in cigar, where consecutive deletions are counted as one.
4, 5, 6, 7	Amount a, c, g, ts	Accumulated numbers of nucleotides in read, separately counted for a, c, g and t
8	Read length	Length of the read
9	Mapping quality	Mapping quality delivered by Minimap 2
10, 11, 12	Insertion length ($1^{st}, 2^{nd}$ & 3rd quartile)	$1^{st}, 2^{nd}$ & 3rd quartile of the distribution of insertion lengths. See diagram: 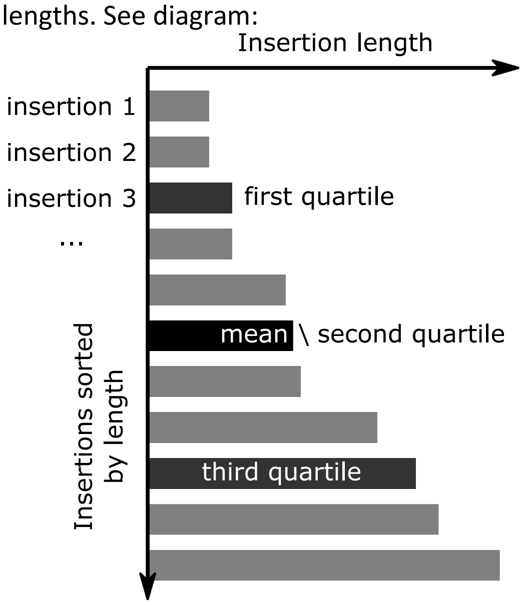
13, 14, 15	Deletion length ($1^{st}, 2^{nd}$ & 3rd quartile)	$1^{st}, 2^{nd}$ & 3rd quartile of the distribution of deletion lengths.
16, 17, 18	Exact match length ($1^{st}, 2^{nd}$ & 3rd quartile)	$1^{st}, 2^{nd}$ & 3rd quartile of the distribution of exact match lengths.
19	Amount exact matches	Accumulated number of exact matches in cigar, where consecutive matches are counted as one.
20	Alignment length	Accumulated alignment length (i.e., assumed the cigar comprises 10 matches and 10 insertions of length 2, then the alignment length is 30).

Supplementary Note 1 - Error profile sampling and read generation

We generate a read length distribution as well as insertion/deletion length distributions for a given dataset (see Supp. Table 2) as follows:
For all reads in the dataset, we store the size of each read in a multiset, which results in a read length distribution. Then, we align each read using Minimap2 and parse the cigars with respect to insertions and deletions. The sizes of all observed insertions are stored in a multiset; this is the insertion length distribution. In the same way, we create a deletion length distribution.
Additionally, the alignments are used for the creation of mean error rates. In order to get these rates, we individually count the total number of substitutions, insertions and deletions within all alignments, where consecutive insertions or deletions are counted as one. Using these counts, we generate the error rates by normalizing with the total number of aligned nucleotides. The resulting three rates are later used during read generation as probabilities for the injection of substitutions, deletions and insertions. In the context of the generation, the size of an injected insertion/deletion is a randomly picked value from the insertion/deletion length distribution. This is justified by Supp. Fig. 3.1 and 3.2 of Sedlazeck et al.[1], where the displayed error rates oscillate around an almost fixed mean. Our error profiles comprise two kinds of information: First, there is the error induced by the sequencer. Second, there is the difference between the sequenced genome and the reference genome. Therefore, the sampled error profiles reflect real world challenges of aligners.

We will now evaluate our read simulation scheme using a principal component analysis (PCA)[2]. For accomplishing the PCA, we use *scikit learn*[3] available for Python 3 (within *scikit learn, the dimensionality reduction library*). The authenticity of simulated reads is assessed in the context of the genome used for read generation. Therefore, we analyze alignments of reads instead of the reads themselves. For alignment computation, we rely on Minimap 2[4] with the appropriate presetting for the respective types of reads (PacBio: *map-pb*; UON: *map-ont*). The provided alignments are placed in a 20-dimensional space, where the individual dimensions are listed in Supplementary Table 3.
Using a principal component analysis (PCA), we reduce the 20-dimensional space down to two dimensions. The PCA determines the dimensions with the highest variances and transforms them in a way, so that differences become well visible in lower dimensions. The PCA is performed on the joint set of original reads and all simulated reads. After PCA application, the points originating from original reads and the points originating from simulated reads are separated and visualized in individual plots. The visual similarity among a pair of plots characterizes the similarity among their reads. If one plot belongs to the original reads and the other belongs to simulated reads, their resemblance indicates the authenticity of the simulated reads.

Supplementary Fig. 4 - Extended accuracy analysis for simulated Illumina reads

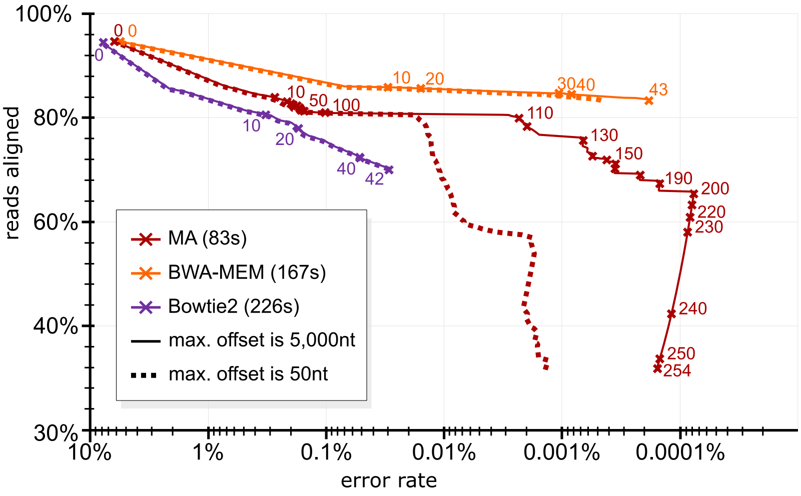

The command line parameter ‘wiggle’ of DWGSIM_EVAL (part of DWGSIM) specifies a maximal offset from the correct position that is tolerated for an alignment to be considered accurate. The above figure compares all three aligners for the maximal offsets 50nt and 5,000nt (dotted lines represent the curves for a maximal offset of 50nt). The graphs for the 50nt offset are identical to the curves shown in Fig. 3 of the manuscript.

Compared to its two competitors, MA shows a particularly high error rate decrease for an offset of 5,000nt. This behavior can be traced back to a characteristic of MA within repetitive regions. For such regions, MA often identifies the region itself but does not recognize its repetitive character. By incorporating an efficient detection mechanism for these cases, MA could reach lower error rates and deliver better mapping quality estimations for this kind of short reads.

The runtimes noted in the legend are measured using the benchmarking tools described in Supp. Note 8. Further, the command line parameters for the individual aligners are listed below:

**Aligner**	**Command line call**
MA	-p Illumina -d 500 -S 50 -i mate1.fasta,mate2.fasta -o out.sam
BWA-MEM	-t 32 mate1.fasta mate2.fasta > out.sam
Bowtie 2	-p 32 -1 mate1.fasta -2 mate2.fasta -S out.sam

Supplementary Fig. 5 - Read generation for injected deletions and insertions with SVs

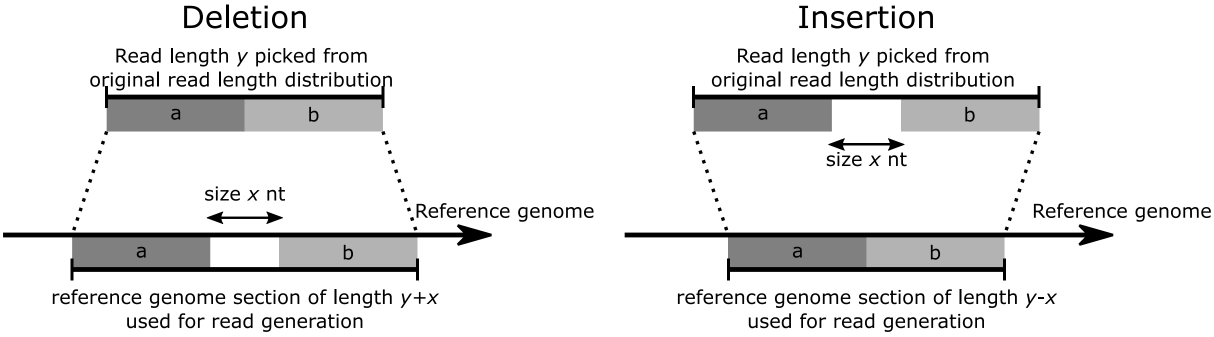

The figure visualizes the scheme for read length adjustment. More details are given in Supp. Note 2.

Supplementary Note 2 - Structural variants

For analyzing SV, we generate 1000 reads for insertions and 1000 reads for deletions with one injected SV per read as follows: We create purged variants of the insertion distribution and deletion distribution, where all indel sizes >10nt are removed from the respective multisets. This purging shall avoid confusion between injected SV indels and indels that originate from the read sampling. Further, we create a purged variant of the read length distribution, where we set all elements to a minimum size of 1000nt. Then, we generate reads using the mean error rates and the purged length distributions as described in Supp. Note 1. Here, we pick a read length adjusted by the size of the SV as shown in Supp. Fig. 5. Finally, we inject one SV of predetermined kind and size into each generated read at the center position. E.g. in the case of a deletion of 1000nt and a picked read length of 3000nt, we first select a random section of 4000nt from the reference genome, modify it as described in Supp. Note 1 and finally delete 1000 consecutive nucleotides in the center.
The generated reads are used for evaluating aligners by analyzing the delivered alignment's CIGARs. Depending on a CIGAR for a read with injected SV, we distinguish among six categories of alignments: precise, split, indicated, forced, trimmed and unaligned. These categories are motivated by Sedlazeck et al.[1]. Supp. Fig. 6 gives a graphical description of these categories.

For insertions, the detailed categories are as follows:

precise: The aligner reports the SV as single insertion within the cigar, where the position of the insertion on the reference genome is within a range of 10nt of the correct position.

split: The aligner splits the insertion by delivering two alignments, where the end-points of these two alignments are within 10nt of the correct position of the SV.

indicated: The aligner recognizes the SV as single insertion within the cigar. The indicated position is not precise, but still within a range of $\pm$100nt of the correct position.

forced: The aligner distributes the SV among several insertions. This is recognized, by checking for an alignment that spans from 100nt before to 100nt after the injected insertion without reporting an insertion $\geq$25nt.

trimmed: The aligner delivers only one alignment with an end-point that is within 100nt of the position of the SV (on reference).

unaligned: The SV stays completely unrecognized by the aligner.

For deletions, the detailed categories are as follows:

precise: The aligner indicates the SV as single deletion within the cigar, where the position of the reported start position and end position are within a range of 10nt of the correct positions on reference.

split: The aligner reports the deletion using two separated alignments, where the end-points of these two alignments are within 10nt of the correct positions (start and end position) of the SV on reference.

indicated: The aligner recognizes the SV as single deletion within the cigar. The indicated positions (start and end position) are not precise, but still within a range of $\pm$100nt of the correct positions.

forced: The aligner distributes the SV among several deletions. This is recognized, by checking for an alignment that spans from 10nt before to 10nt after the injected deletion without reporting a deletion $\geq$25nt.

trimmed: Either, the aligner delivers an alignment with an endpoint that is within 100nt of the start of the SV (on reference) or the aligner delivers an alignment with a start point that is within 100nt of the end of the SV.

unaligned: The SV stays completely unrecognized by the aligner.

We check alignments in the order forced, precise, split, indicated, trimmed, unaligned and classify an alignment by the first matching category. Please note: classifying alignments using the above order implies that split alignments, where at least one CIGAR-end is not within 10nt distance (but within 100nt) from the injected SV, are considered “trimmed”. In the context of the CIGAR analysis, we ignore all indels of size <25nt because indels of this size are likely to be a side effect of the indel length distributions. The SV information is lost with the trimmed and unaligned categories, while SV callers might be able to reconstruct the correct SV from split, indicated and forced alignments. Nonetheless, precisely aligned reads are best, as they accurately indicate the correct SV.

Supplementary Fig. 6 - SV mapping for PacBio reads

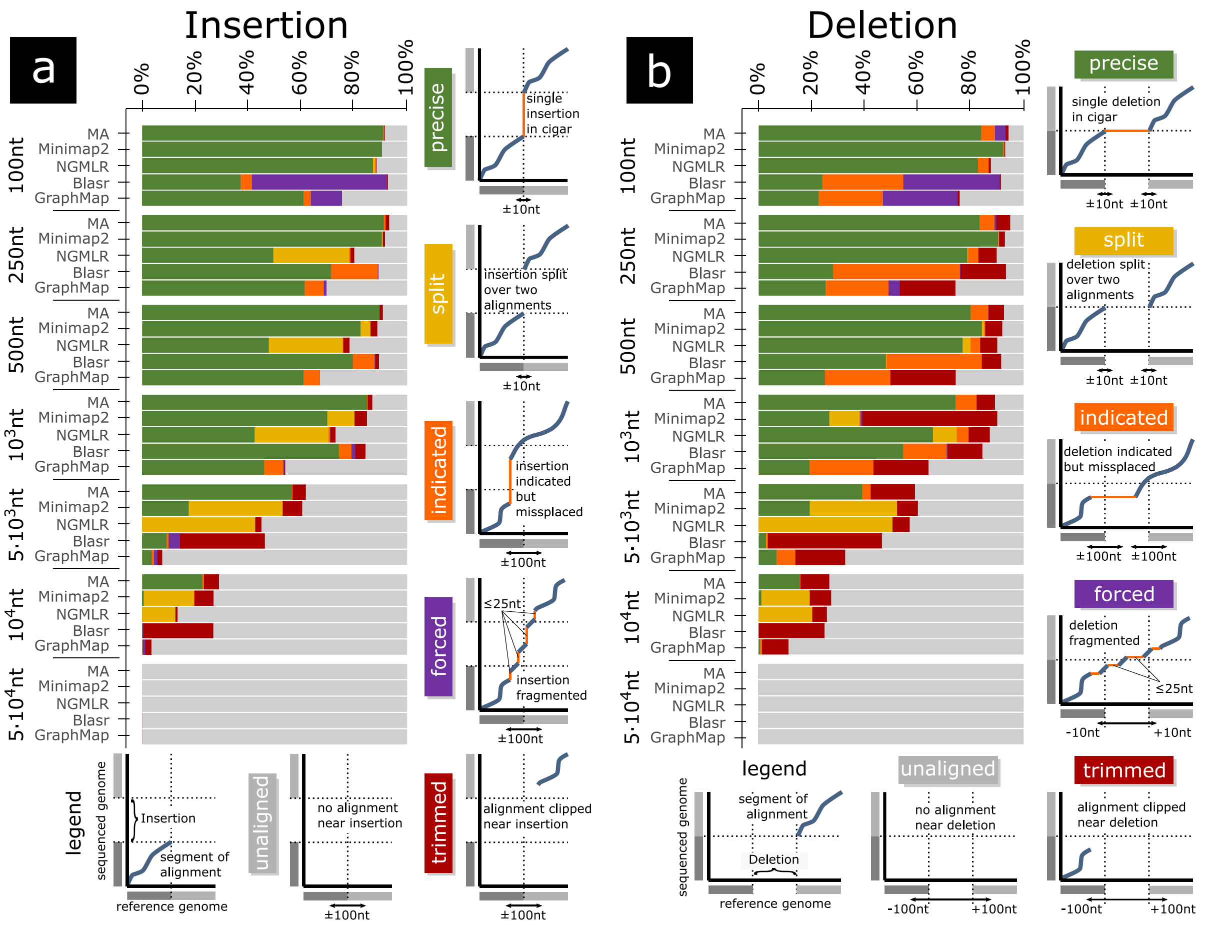

The following mode/presetting is used for the computation of the diagrams:
MA: “fast”; Minimap2: “map-pb”; NGMLR: “pacbio”. For all other aligners: the default mode is used.

Supplementary Note 3 - Maximally spanning seeds

MAXIMAL-EXTEND$(d,\ I_R,\ Q,\ A,\ i,\ s)$
1 **while** $i \geq 0$ **and** $i < |Q|$
2 **do** $A' \leftarrow I_R^d(Q[i],\ A)$ ▻ compute new seed set
3 **if** $A' \neq \emptyset$ ▻ use the new set if it is not empty
4 $A \leftarrow A'$
5 **if** $d =$ "←"
6 $s \leftarrow Q[i] \oplus s$
7 $i \leftarrow i - 1$
8 **else** $s \leftarrow s \oplus Q[i]$
9 $i \leftarrow i + 1$
10 **else break**
11 **return** Tuple$(A,\ s)$

MAXIMALLY-SPANNING$(I_R,\ Q,\ i)$
1 $A \leftarrow I_R^*(Q[i])$
2 $(A',\ s) \leftarrow$ MAXIMAL-EXTEND("←", $I_R,\ Q,\ A,\ i-1,\ Q[i]$)
3 $(A',\ s) \leftarrow$ MAXIMAL-EXTEND("→", $I_R,\ Q,\ A',\ i+1,\ s$)
4 $(A,\ s) \leftarrow$ MAXIMAL-EXTEND("→", $I_R,\ Q,\ A,\ i+1,\ Q[i]$)
5 $(A,\ s) \leftarrow$ MAXIMAL-EXTEND("←", $I_R,\ Q,\ A,\ i-1,\ s$)
6 **return** $A \cup A'$

MAXIMALLY-SPANNING computes maximally spanning seeds. Two seed sets are discovered:

- One seed set is computed by a maximal backwards extension (line 2) followed by a maximal forwards extension (line 3).
- The second seed set is computed by a maximal forwards extension (line 4) followed by a maximal backwards extension (line 5).

Both extensions start with the nucleotide at position i. d (direction) indicates whether a maximal forwards ("→") or backwards ("←") extension shall be performed.

Supplementary Fig. 7 - Seed ambiguity analysis

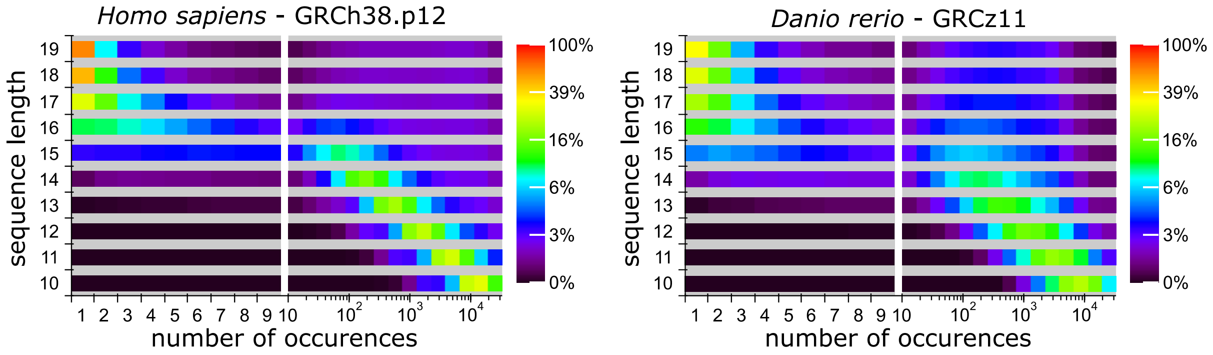

The diagram shows the distribution of the number of occurrences for 10nt to 19nt sequences on the human genome. E.g. the light orange box for x=1, y=18 indicates that roughly 40% of all sequences of length 18 occur exactly once on the human genome. The percentage values of each row are computed by picking 1000 unique sequences from random locations on the genome and counting the number of occurrences individually for each sequence.

In the context of k-mers of size 15, the diagram comprises the information that the majority of seeds is expected to occur on roughly 60 to 70 different positions on the human genome. Roughly 1% of all seeds are expected to occur on 10^4 positions or even more.

Fixed-sized seeding techniques require adaptions with respect to the sequencer technology as well as the genome. For example, Minimap2 choses a k-mer size of 15 for the setting “map-ont”. Minimap2 choses this low k-mer size in order to get enough seeds for the noisy Ultralong Oxford Nanopore reads (the setting “map-pb” for PacBio reads uses a seed size of 19). For the human genome, this k-mer size does not imply trouble with multiple occurrences of seeds because the majority of seeds occurs less than 200 times on the genome. However, for *Danio rerio* the distribution is less fortunate.

Supplementary Note 4 - Binary seeding

BINARY-SEEDING(I_R, Q, b, e)
if $e \leq b$
return $\emptyset$
$S \leftarrow$ MAXIMALLY-SPANNING(I_R, Q, $\lfloor (b + e)/2 \rfloor$)
$S' \leftarrow$ BINARY-SEEDING(I_R, Q, b, S.begin)
$S'' \leftarrow$ BINARY-SEEDING(I_R, Q, S.end, e)
return $S' \cup S \cup S''$

BINARY-SEEDING computes a fully covering set of seeds. It relies on MAXIMALLY-SPANNING in order to obtain seeds that overlap a given index position. Initially, b must be 0 and e must be $|Q|$.

Supplementary Fig. 8 - Unrelated Seeds

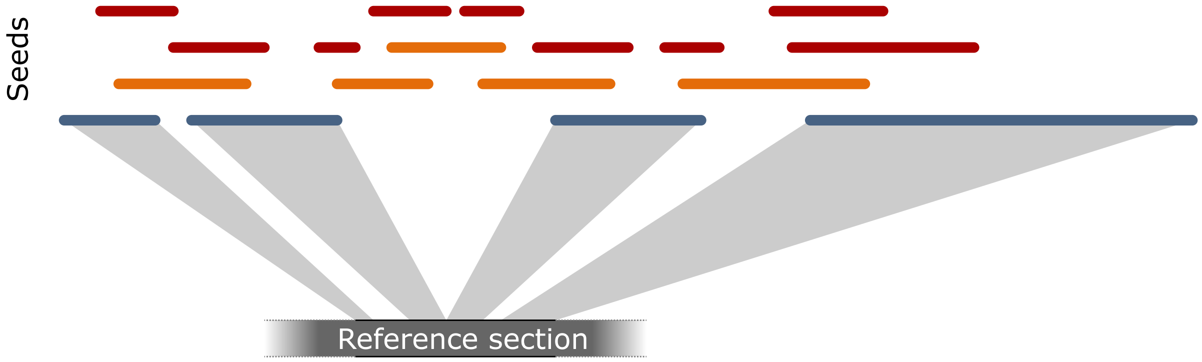

The figure shows an alignment scenario with several seeds. The blue seeds are part of the optimal alignment; these seeds are called relevant seeds. Their positions on the reference are indicated by grey polygons. The red and orange seeds are irrelevant. Red seeds can be recognized, since they are completely covered by longer seeds. It is difficult to distinguish blue and orange seeds during seeding, because there is no trivial relationship between seed size and seed relevance. Seeding via SMEMs comprises all shown seeds. Only 4.4% (4.9%) of all red seeds are collected with the maximally spanning extension scheme with the UON (PacBio) dataset. Maximally spanning seeds comprise all blue and orange seeds.

Supplementary Fig. 9 - Maximally spanning seeds and SMEMs on long reads

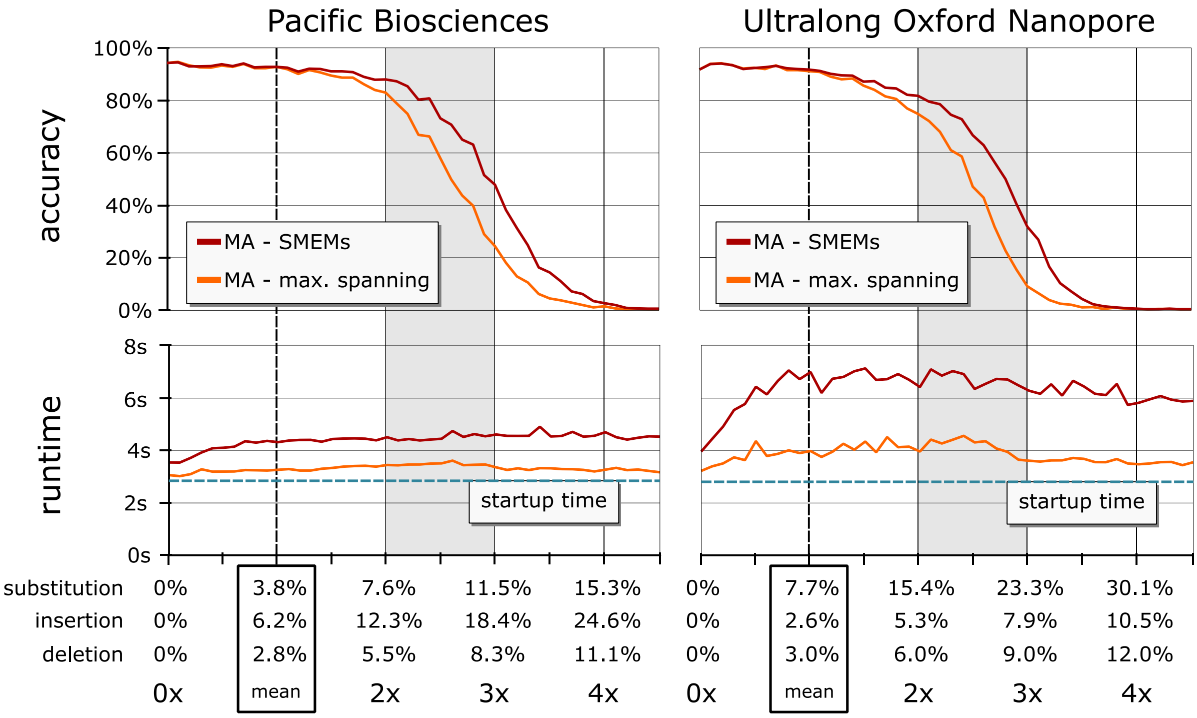

The above figure details the runtimes and accuracy of Fig. 2 in the manuscript by looking into MA's seeding modes (SMEMs and maximally spanning seeds) individually. Additionally, MA's startup time is indicated by a blue dashed line. The diagrams show that up to the mean error, there is no difference between both seeding techniques with respect to accuracy. However, in this range, SMEMs inflict a needless runtime penalty due to the larger number of seeds compared to maximally spanning seeding. The higher quantity of SMEMs is only advantageous over the higher quality of maximally spanning seeds for noisy reads (2 times up to 4 times the mean error). With the inspected datasets for PacBio and UON only a small percentage of all reads is within this critical range.

Supplementary Note 5 - Width of Strip of Consideration

If the gap between two seeds results in a penalty that exceeds the maximal alignment score (with respect to the query and a given score for matches), then both seeds cannot be part of the same local alignment. The size of the SoC should be chosen so that we collect all spatially local seeds. Let s_{M} be the score for a match and let $p_{\mathrm{O}}, p_{\mathrm{E}}$ be the penalties for opening and extending a gap, which are chosen according to the dynamic programming scoring scheme. We now compute the optimal SoC width:

We consider two seeds $s = (q, r, l)$ and $s' = (q', r', l')$ and a query Q. If s and s' shall belong to the same SoC we must have:

$$\text{gap-cost}(s, s') < \text{max-score}(|Q|)$$
$$= p_{\mathrm{O}} + p_{\mathrm{E}} \cdot |d_Q - d_R| < s_{\mathrm{M}}|Q| \quad (1)$$

d_Q in the above formula is the distance between s and s' on the query and d_R is the distance between s and s' on the reference. Using Supp. Fig. 10, we can see that $|d_Q - d_R|$ must be filled using one or multiple gaps. Due to the nature of affine gap costs, one single gap of size x is always cheaper than multiple gaps that sum-up to size x (or of equal cost if the gap open penalty is zero). We have $\delta := r - q$ and $\delta' := r' - q'$. δ and δ' represent SoC locations as shown in Supp. Fig. 10. In order to simplify equation (1), we use the following:

Lemma 1: $|d_Q - d_R| = |\delta - \delta'|$

Proof: (1) Assume s is to the bottom left of s' ($q < q' - l; r < r' - l$). Then

$$\begin{aligned}&|d_Q - d_R|\\ &= |(q - q' - l) - (r - r' - l)|\\ &= |-(q - q' - l) + (r - r' - l)|\\ &= |(r - q) - (r' + l - q' - l)|\\ &= |(r - q) - (r' - q')|\\ &= |\delta - \delta'|.\end{aligned}$$

(2) Assume s is to the top right of s' ($q' < q - l'; r' < r - l'$). Then

$$\begin{aligned}&|d_Q - d_R|\\ &= |(q' - q - l') - (r' - r - l')|\\ &= |(q' - q) - (r' - r)|\\ &= |(r - q) - (r' - q')|\\ &= |\delta - \delta'|.\end{aligned}$$

(3) In all other cases either d_Q or d_R is not given. ■

We can now derive the optimal SoC width from Supplementary Equation 1 in combination with Lemma 1:

$p_{\mathrm{O}} + p_{\mathrm{E}} \cdot |d_Q - d_R| = p_{\mathrm{O}} + p_{\mathrm{E}} \cdot |\delta - \delta'| < s_{\mathrm{M}}|Q|$.

Therefore, we have

$$|\delta - \delta'| < \frac{s_{\mathrm{M}}|Q| - p_{\mathrm{O}}}{p_{\mathrm{E}}}.$$

Hence, two seeds need to be closer than $\frac{s_{\mathrm{M}}|Q| - p_{\mathrm{O}}}{p_{\mathrm{E}}}$ to be considered spatially local. So, the optimal width of a SoC is $\frac{s_{\mathrm{M}}|Q| - p_{\mathrm{O}}}{p_{\mathrm{E}}}$ which we denote by λ.

Supplementary Fig. 10 - Gaps between seeds

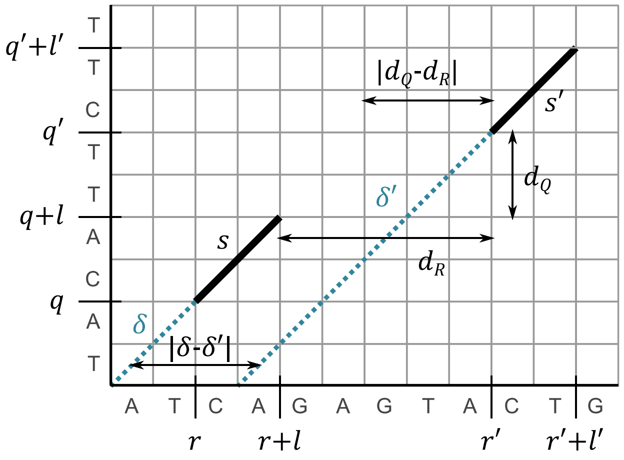

Gaps of cumulative length $\geq |d_Q - d_R|$ are required in order to fill the space between s and s'.

Supplementary Note 6 - Strip of consideration computation

```
SoC(S)                                                   ▻ S is a vector of triples (δ, l), where each
                                                           triple represents a seed
1    SORT(S) ascending using the first element
2    let M be an empty stack of tuples (start, score)
3    x ← 0;  b ← 0;  e ← 0;  δ' ← 0;  x' ← 0
4    while e < |S|                                       ▻ move scanline along sorted seeds
5        do while e < |S| and S[b].δ + λ ≥ S[e].δ        ▻ move e forward
6            do x ← x + S[e].l                           ▻ add score of current seed
7               e ← e + 1                                ▻ jump to next seed (SoC end)
8            if |M| = 0 or δ' < S[b].δ or x' < x         ▻ check for already pushed SoCs with
                                                           higher score
9               if |M| > 0 and δ' ≥ S[b].δ
10                  M.pop()                              ▻ replace previous SoC instead of inserting
                                                           a fresh one
11              M.push( Tuple(b, x) )
12              δ' ← S[e].δ; x' ← x
13           x ← x − S[b].l                              ▻ remove score of current seed
14           b ← b + 1                                   ▻ jump to next seed (SoC begin)
15   return PriorityQueue(M)                             ▻ transform into a priority queue using
                                                           BUILD-MAX-HEAP
```

The above Pseudocode describes the computation of Strip of Considerations (SoC). S is a vector of seeds. The code decomposes into three sections:

- An initializing step (line 1-3) sorts the incoming seeds according to their δ-values. Further, it initializes a stack of tuples and several variables. All this can be done in time $O(n \log n)$, where n is the number of seeds in S.
- A collection of non-overlapping SoC happens in the lines 4-16. The code block follows the scanline principle and requires time $O(n)$.
- Finally, the SoCs are turned into a priority queue using the BUILD-MAX-HEAP procedure. The construction of the priority queue can be done in time $O(n)$.

If the vector S is already sorted, the time complexity of SOC is $\Theta(n)$. In all other cases the worst-case complexity is $O(n \log n)$.

Supplementary Note 7 - Seed harmonization

```
LINE-SWEEP(V, δ)                                      ▷ V is a vector of triples (seed, begin
                                                        shadow, end shadow); δ is the alignment
                                                        guideline
  SORT(V) ascending using the shadow begin
        for equal beginnings sort descending
        using the shadow end
  let T be an empty vector of tuples                  ▷ (seed, end of shadow)
  for each (s, b, e) in V                             ▷ Iterate over the sorted seeds.
      i ← |T| − 1
      if i < 0 or T[i].second < e                     ▷ The end positions have the correct
          T.push_back( Tuple(s, e) )                    order: no contradiction
      else while i > 0 and T[i].second ≥ e            ▷ There is a contradicting seed
              do if DIST(T[i].first, δ) ≥ DIST(s, δ)  ▷ Check if seed is closer to δ
                      i ← i − 1                       ▷ we found such a seed
                  else i ← |T|; break
          T ← T[0, i)                                 ▷ If there is no such seed remove all
  return T                                              contradicting seeds; otherwise remove
                                                        the current seed (implicit)
```

```
HARMONIZATION(S)                                      ▷ S is a vector of triples (q, r, l),
  let V_II, V_IV be empty vectors of triples            where each triple represents a seed
        (seed, shadow begin, shadow end)
  δ ← RANSAC(S)                                       ▷ compute the δ-guideline
  for s in S with s = (q, r, l)                       ▷ compute σ_II
      V_II.append( Tuple(s, q, r + l) )
  for (s, e) in LINE-SWEEP(V_II, δ) with s = (q, r, l) ▷ purge seeds contradicting in σ_II
      V_IV.append( Tuple(s, r, q + l) )                 and compute σ_IV
  return LINE-SWEEP(V_IV, δ)                          ▷ purge seeds contradicting in σ_IV
```

HARMONIZATION(S) purges the set of seeds S with respect to contradicting seeds. For this purpose, it calls the function LINE-SWEEP twice. LINE-SWEEP comprises a solution for the interval-interval inclusion problem. The interval-interval inclusion problem is the problem of identifying fully enclosed intervals in a set of intervals. LINE-SWEEP(V, δ) purges all enclosed intervals (each enclosed interval represents a contradicting seed) in V.

Supplementary Note 8 - Benchmarking tools

All runtimes are measured on an *AMD Ryzen Threadripper 1950X 16-Core Processor* with 128 GB RAM.

The maximal RAM consumption reported in Table 1 of the manuscript is measured using valgrind[5] (*valgrind --tool=massif --pages-as-heap=yes*), based on simulated PacBio reads for long read aligners (incl. MA) and simulated Illumina reads for short read aligners.

Via command line parameters, benchmarked aligners had been configured to exploit all 32 virtual cores of the AMD Ryzen processor.

Supplementary References

1 Sedlazeck, F. J. *et al.* Accurate detection of complex structural variations using single-molecule sequencing. *Nature Methods* **15**, 461-468, doi:10.1038/s41592-018-0001-7 (2018).
2 Jolliffe, I. in *International encyclopedia of statistical science* 1094-1096 (Springer, 2011).
3 Pedregosa, F. *et al.* Scikit-learn: Machine learning in Python. *Journal of machine learning research* **12**, 2825-2830 (2011).
4 Li, H. Minimap2: pairwise alignment for nucleotide sequences. *Bioinformatics* **1**, 7 (2018).
5 Nethercote, N. & Seward, J. in *ACM Sigplan notices.* 89-100 (ACM).
